# The Effect of Limiting the Scan Range of Computed Tomography Pulmonary Angiography (to Reduce Radiation Exposure) on the Detection of Pulmonary Embolism: A Systematic Review

**DOI:** 10.3390/diagnostics11122179

**Published:** 2021-11-24

**Authors:** Amayar Zaw, Rebecca Nguyen, Leon Lam, Anthony Kaplan, Claudia C. Dobler

**Affiliations:** 1 Department of Respiratory and Sleep Medicine, Liverpool Hospital, Sydney 2107, Australia; amayarzaw24@gmail.com (A.Z.); rebecca.han.nguyen@gmail.com (R.N.); 2 South Western Sydney Clinical School, University of New South Wales, Sydney 2107, Australia; anthony.kaplan@health.nsw.gov.au; 3 Department of Radiology, Liverpool Hospital, Sydney 2107, Australia; leon.lam@health.nsw.gov.au; 4 The George Institute for Global Health, Sydney 2042, Australia; 5 Woolcock Institute of Medical Research, The University of Sydney, Sydney 2037, Australia

**Keywords:** pulmonary embolism, diagnostic imaging, computed tomography pulmonary angiography radiation, cohort studies, systematic review

## Abstract

(1) Background: Computed tomography pulmonary angiography (CTPA) is the standard imaging test for the evaluation of acute pulmonary embolism (PE), but it is associated with patients’ exposure to radiation. Studies have suggested that radiation exposure can be reduced without compromising PE detection by limiting the scan range (the *z*-axis, going from up to down); (2) Methods: A literature search was conducted in MEDLINE and EMBASE on 17 July 2021. Studies were included if they enrolled patients who had undergone a CTPA and described the yield of PE diagnoses, number of missed filling defects and/or other diagnoses using a reduced *z*-axis in comparison to a full-length scan. To assess risk of bias, we modified an existing risk of bias tools for observational studies, the Newcastle-Ottawa Scale. Results were synthesized in a narrative review. Primary outcomes were the number of missed PE diagnoses (based on at least one filling defect) and filling defects; the secondary outcome was the number of other missed findings; (3) Results: Eleven cohort studies and one case-control study were included reporting on a total of 3955 scans including 1025 scans with a diagnosis of PE. Six different reduced scan ranges were assessed; the most studied was from the top of the aortic arch to below the heart, in which no PEs were missed (seven studies). One sub-segmental PE was missed when the scan coverage was 10 cm starting from the bottom of the aortic arch and 14.7 cm starting from the top of the arch. Five studies that reported on other findings all found that other diagnoses were missed with a reduced *z*-axis. Most of the included studies had a high risk of bias; (4) Conclusions: CTPA scan coverage reduction from the top of aortic arch to below the heart reduced radiation exposure without affecting PE diagnoses, but studies were generally at high risk of bias.

## 1. Introduction

Pulmonary embolism (PE) is a common and treatable disease that, if missed, can be associated with high morbidity and mortality, making efficient and accurate diagnosis important to ensure proper patient care [1]. Definitive diagnosis is achieved with the current diagnostic gold standard—computed tomography pulmonary angiography (CTPA) [2]. Relative to other imaging modalities, CTPAs are fast and minimally invasive, highly specific for PE and widely accessible. They are a frequently ordered test to diagnose or exclude PE. In countries such as the United States of America, millions of CTPAs are performed every year [3], but less than 10% of scans performed return positive results [4]. The relatively low yield of the diagnostic test highlights the importance of clinical risk scores to help identify patients with high pre-test probability for PE, but also the importance of weighing up risks and benefits of the imaging test. The most significant risk is the exposure to ionizing radiation. This is of particular concern in young and/or pregnant women, because of a potential long-term increased risk of breast cancer and possible harm to the fetus [5]. Additionally, there is a cumulative risk from repeat scans, as one-third of patients who undergo a CTPA for the first time will undergo a second CTPA within the subsequent five years [6]. Widespread usage of CTPA is also associated with an increased rate of detection of non-PE findings. While identification of other pathologies can be beneficial, for example when identifying alternative etiologies of a patient’s symptoms, discovery of incidental findings can also lead to potentially unnecessary further diagnostic investigations and treatments [7].

The widespread use of CTPA warrants careful consideration of strategies to reduce radiation exposure-related risks to patients. Reducing the CTPA *z*-axis (the axis going from up to down) reduces radiation exposure without impairing image quality. As PEs are most commonly found in the lobar and segmental pulmonary arteries in the central portions of the scan [8], there is potential to reduce the boundaries of a scan, such as excluding the lung apices and sub-diaphragmatic regions, while maintaining diagnostic accuracy. However, this would also exclude peripheral areas of the lung containing sub-segmental arteries. As the benefit of treating isolated sub-segmental PEs are unclear [9], it might be reasonable to exclude these regions in order to focus on capturing clinically significant PE that do require treatment.

This is the first systematic review of the effect of a limited CTPA scan coverage on the diagnostic yield for PE and other pathological findings. The goal is to inform clinical practice regarding the utility of reduced CTPA scan coverage in patients in whom reduced radiation exposure is an important consideration.

## 2. Materials and Methods

This protocol adheres to the Preferred Reporting Items for Systematic reviews and Meta-Analyses (PRISMA) guidelines [10].

### 2.1. Study Selection

Studies were included if they enrolled patients who had undergone a CTPA and described the yield of PE diagnoses, number of missed filling defects and/or other diagnoses using a reduced CTPA *z*-axis in comparison to a full-length CTPA scan. We included all publication types, including full text studies, conference abstracts or correspondence with original data in any language. Studies were excluded if they were case reports with fewer than five cases, systematic reviews or narrative reviews.

### 2.2. Literature Search

A systematic search of MEDLINE and EMBASE was performed on 17 July 2021. The search strategy can be found in Table S1. After the removal of duplicates, two reviewers (A.Z. and R.N.) independently assessed the titles and abstracts of all the studies identified by the search against the study selection criteria. For all studies included by either reviewer, the full-text versions were then screened for a final decision about eligibility. The reference lists of the full texts and any identified reviews were examined to find additional relevant studies not identified by the search strategy. In the case of unresolved discrepancies, a third reviewer (C.C.D.) resolved any disagreements.

### 2.3. Data Extraction

We created a data extraction sheet, which was pilot-tested with three studies. A number of modifications were subsequently made to the extraction sheet to ensure all relevant information was captured. Two reviewers (A.Z. and R.N.) extracted all data independently. Results were compared and any discrepancies were discussed and resolved, if possible. Any unresolved discrepancies were resolved by a third reviewer (C.C.D.).

### 2.4. Quality Assessment

To assess risk of bias, we modified an existing risk of bias tools for observational studies, the Newcastle-Ottawa Scale, to allow for the fact that the study “subjects” were CTPA scans rather than people (see Table S2). The maximum score per study (representing the lowest risk of bias) was eight points across two domains. First, we assessed the sample size and the proportion of excluded CTPA scans due to image quality (e.g., sub-optimal artery opacification and breathing artefacts). This evaluated the generalizability of the findings to all CTPA scans ordered in clinical practice and also appraised the robustness of the study results. Secondly, we assessed the quality of the process undertaken to analyze the CTPAs—the number of reviewers, their experience and the process of blinding, if any.

### 2.5. Outcome Measures

The primary outcomes measured were the number of PE diagnoses (based on at least one filling defect) and the number of filling defects missed with a reduced CTPA *z*-axis compared to a full-length CTPA. The secondary outcome measured was the number of other non-PE diagnoses missed with the same reduced scan window compared to a full-length CTPA.

## 3. Results

**Figure 1:**
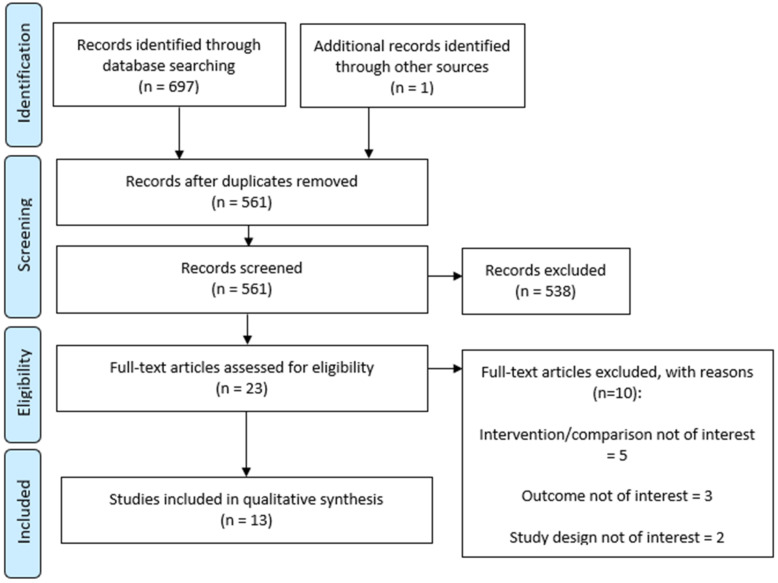
PRISMA flow diagram.

The search strategy identified 563 unique citations, of which 23 studies were included in the full-text screening (Figure 1). Thirteen studies met the inclusion criteria—12 were directly identified by the search algorithm and an additional study was identified via the reference list of one of the included studies. An overview of the studies excluded after full-text screening can be found in Table S4. All included studies were observational studies. Eight studies were published as peer-reviewed articles and five as conference abstracts.

The characteristics of the included studies are summarized in Table 1, and the CTPA technical parameters and boundaries for the reduced scan window used by each study are described in Table 2. The studies were conducted in the United States of America [11,12,13,14,15] (n = 5), Japan [16] (n = 1), England [17] (n = 1), Belgium [18] (n = 1), Australia [19] (n = 1) and the Netherlands [20] (n = 1), and the country location of three studies was not specified [21,22,23]. Eleven studies were conducted in a single hospital (including one in a pediatric center [21]). Of these, two were performed across emergency department (ED), in-patient and out-patient settings [12,15]; one was performed in both ED and in-patient settings [18]; two were performed in an ED setting only [11,14]; and six studies did not specify the patient setting [13,16,17,19,22]. Two studies did not specify the type of institution or setting [20,23].

**Figure 2:**
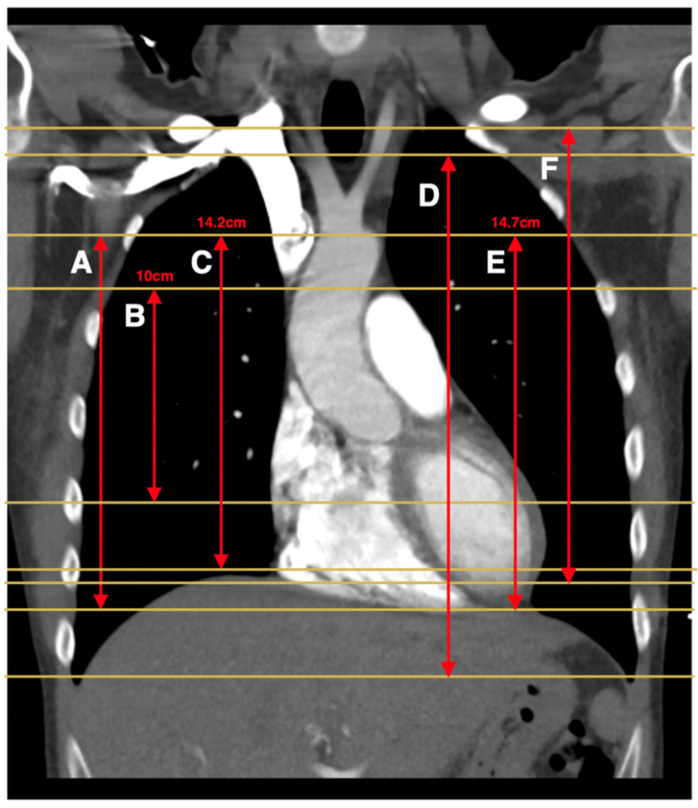
CTPA *z*-axis coverages assessed by studies: (**A**) top of the aortic arch to below the heart; (**B**) 10 cm window starting from the bottom of the aortic arch; (**C**) 14.2 cm window centered 4.1 cm below the carina; (**D**) humeral heads to lung bases (excluding apices); (**E**) 14.7 cm window starting from the top of the aortic arch; (**F**) top of the lung apices to most caudal diaphragm.

Six different reduced CTPA z-axes were pre-defined and assessed in the included studies, as depicted in Figure 2. Seven studies used reduced *z*-axis coverage from the top of the aortic arch extending to below the heart (window A in Figure 2) [11,12,13,15,16,22,23]. One study analyzed a 10 cm *z*-axis length beginning superiorly from the bottom of the aortic arch (window B in Figure 2) [18]. One study used a 14.2 cm *z*-axis length centered 4.1 cm below the carina (window C in Figure 2) [14]. One study used a reduced scan window from the humeral heads to the lung bases, excluding the lung apices (window D in Figure 2) [17]. One study used a 14.7 cm scan length starting at the top of the aortic arch (window E in Figure 2) [19]. Finally, one study used a scan window from the lung apices to the most caudal diaphragm (window F in Figure 2) [20].

Additionally, two studies retrospectively determined the minimum optimal scan window to capture all PE diagnoses and/or filling defects, based on a dataset of CTPA scans which were positive for PE [11,21]. One study assessed 100 consecutive CTPA scans positive for PE and found that a 14.2 cm *z*-axis length centered 4.1 cm below the carina captured at least one filling defect (i.e., all PE diagnoses), and an 18 cm scan length centered 4 cm below the carina captured all filling defects [11]. The second study included 45 CTPA scans positive for PE in patients aged 0–25 years and found that an 8 cm *z*-axis length centered 5 cm below the carina captured at least one filling defect, and a 14 cm *z*-axis length centered 3.5 cm below the carina captured all filling defects [21].

Of the 12 studies that reported the yield of PE diagnoses and/or filling defects with a pre-defined reduced scan window [11,12,13,14,15,16,17,18,19,20,22,23], two studies each reported one case of missed PE diagnosis (based on at least one filling defect) [18,19] (Table 3). Specifically, in one study, one diagnosis of a sub-segmental PE out of 57 PE diagnoses (1.8%) was missed when the *z*-axis was reduced to a 10 cm window starting from the bottom of the aortic arch [18], and in another study, one sub-segmental PE out of 200 PE diagnoses (0.5%) was missed when a 14.7 cm *z*-axis starting from the top of the aortic arch was used [19]. No PE diagnoses were missed when the *z*-axis was reduced from the top of the aortic arch to the below of the heart [11,12,13,15,16,20,22,23], from the humeral heads to the lung bases [17] or when a 14.2 cm *z*-axis was centered 4.1 cm below the carina [14].

Of the three studies [11,16,19] that reported on the diagnostic yield of their reduced scan windows in capturing all filling defects, two studies reported instances of missed filling defects. In one study, 2.7% of filling defects (n = 2) were missed in two patients when the *z*-axis was reduced to a *z*-axis from the top of the aortic arch to under-the-surface of the heart; however, the diagnosis of PE was not missed due the presence of other filling defects within the scan field [16]. In the second study, 1% of filling defects (n = 2) were missed in two patients using a fixed 14.7 cm *z*-axis starting from the top of the aortic arch [19]. Of the two filling defects missed, one was located outside the reduced scan window and resulted in a missed diagnosis of PE [19].

Six studies reported on the yield of diagnoses other than PE; all found that other diagnoses were missed when the scan window was reduced (Table 3) [12,14,15,18,20,22]. When the scan window covered the area from the top of the aortic arch to below the heart, the number of missed findings was as follows: 7 out of 392 other findings missed (1.8%) [15], 4 out of 76 other findings missed (5.3%) [12], and one study reported that other findings were missed in three patients without specifying the number of missed diagnoses [22]. When the scan window reached from the lung apices to the most caudal diaphragm, four other findings were missed [20]. When the scan window was limited to a 10 cm scan length beginning at the bottom of the aortic arch, 48 of 343 (14%) other findings were missed [18]. When the scan window was limited to a 14.2 cm scan length centered 4.1 cm below the carina, 63 of 604 (10.4%) other diagnoses were missed [14]. The most common other diagnoses missed were thyroid nodules (n = 27), hiatal hernia (n = 21) and cholelithiasis (n = 12), which were likely not associated with the patients’ symptoms at presentation.

Most of the included studies had a high risk of bias (Table S3). Using our modified risk of bias tool, three studies scored 6 or more points and were categorized as having a low risk of bias [12,15,18], and ten studies scored less than 6 points and were categorized as having a high risk of bias [11,13,14,16,17,19,20,21,22,23].

## 4. Discussion

This is the first systematic review of the utility of reduced CTPA *z*-axis for the diagnosis of PE. Of the different types of *z*-axis length assessed, from the top of the aortic arch to below the heart was the most frequently investigated by seven studies, and no PE diagnoses were missed with this *z*-axis length. Diagnosis of a sub-segmental PE was missed once with two different reduced *z*-axis windows—a 10 cm window starting from the bottom of the arch and a 14.7 cm window starting from the top of the aortic arch. Although two studies reported missing filling defects, in one of these studies, despite missing two (2.7%) filling defects, no PE diagnoses were missed due to the presence of additional diagnostic filling defects within the reduced scan window [16]. In all studies that reported on the yield of other diagnoses, other diagnoses were missed regardless of the type of reduced *z*-axis coverage.

Both PE diagnoses that were missed when using different z-axes with an absolute length were isolated sub-segmental PE. The benefit of treating isolated sub-segmental PEs is unclear [24]. The guidelines of the American College of Chest Physicians, for example, suggest that it might be reasonable not to anticoagulated a patient with an isolated sub-segmental PE if there is no evidence of proximal lower extremity deep vein thrombosis or evidence of thrombus elsewhere (e.g., upper extremity clot) and the risk of recurrence is considered low [25].

The major benefit derived from reducing the anatomical scan window is the reduced radiation compared to a full-length scan. While estimating the exact radiation dose reduction is complex due to variability based on different tissue densities, the effective radiation dose is effectively directly proportional to scan length [14]. Since areas such as the lung are impacted less by radiation than tissue-dense areas such as the abdomen [14], reducing the scan window to exclude such areas, which is achieved by all scan windows assessed in our systematic review, greatly reduces radiation exposure. While this is beneficial for all patients, young and pregnant women stand to benefit the most. Bismuth breast shields can reduce radiation dosage by 26–41% [5], but have varied use internationally—within the United States of America, for example, these shields are not routinely used in practice [26]. For a fetus, the radiation dosage associated with a mother’s CTPA is significantly less than the levels required to produce teratogenic effects [27]; however, little is known about the long-term risk and potential genetic damage induced [28]. While in general, the benefits of CTPA seem to outweigh the associated risks in patients with a high pre-test probability of PE, it is desirable to minimize radiation exposure associated with CTPAs, especially in pregnant women, for example by using a reduced anatomical scan window.

Some incidental findings were missed with limited CTPA scan coverages. While some CTPA findings other than PE may offer an alternative diagnosis for the patient’s presentation (e.g., pleural effusion), incidental findings on CTPAs (e.g., of a pulmonary or thyroid nodule) can also lead to over-investigation and over-treatment, which potentially exposes the patient to unnecessary risks [29]. A reduced detection rate of incidental findings on CTPA is therefore not a reason not to use a reduced anatomical cover scan.

This systematic review included two studies [11,21] that did not use predefined anatomical landmarks to reduce the scan window but evaluated the optimal scan range measured in centimeters to capture all identified PEs and/or filling defects. The optimized scan windows that captured at least one filling defect were a 14.2 cm scan length centered 4.1 cm below the carina (based on adult patients) [11] and an 8 cm scan length centered 5 cm below the carina (based on pediatric patients) [21]. The former scan window was subsequently validated in a second study (also included in this systematic review), which found that no PE diagnoses were missed when this scan window was used [14]. Scan windows based on absolute measures and not easily identifiable anatomical landmarks are potentially problematic in clinical practice. Firstly, radiographers routinely use anatomical landmarks to determine the CTPA window [30]. Secondly, absolute measures may not be applicable to all population groups, as patients may have significant variations in anatomy and thorax length. These variations may be due to patient sex, size and underlying medical conditions (such as chronic obstructive pulmonary disease). A *z*-axis defined by anatomical landmarks at both ends is generally applicable independently of the individual’s thorax length. Furthermore, both studies that evaluated the optimal scan range measured in centimeters were performed at a single institution; one study included only adult ED patients [11] and one study included only pediatric patients [21]. Therefore, these results cannot be generalized to other populations.

This study has several limitations. Most of the studies had a high risk of bias. Of the included studies published in peer-reviewed journals, five of eight studies did not specify whether scans were excluded for poor image quality [11,13,14,16,20]. Only three studies specified that radiologists were blinded to the original CTPA report [12,15,18], and one study did not indicate the years of experience of the reporting doctor [16].

Five of the twelve included studies were conference abstracts [17,19,21,22,23]. Conference abstracts are characterized by a dearth of detailed information due to the restricted format as well as lack of peer review when compared to studies published in peer-reviewed journals. None of the included abstracts provided any details pertaining to the items in our risk of bias assessment, and hence, all scored zero points (high risk of bias).

Most of the proposed *z*-axis coverages were only assessed in one study each with a relatively low sample size of CTPAs. However, the CTPA scan coverage from the top of the aortic arch to below the heart was assessed in seven studies and did not miss any PE diagnoses.

## 5. Conclusions

This systematic review demonstrates that there is potential to limit the scan window of a CTPA without reducing the diagnostic yield for PE. Physicians should consider utilizing the option to limit the scan coverage of a CTPA, particularly in population groups where radiation exposure is of special concern, such as pregnant women. The scan coverage from the top of the aortic arch to below the heart may be the best approach in achieving radiation dose reduction without impairing diagnostic yield.

## Figures and Tables

**Table 1:** Study characteristics of included studies.

Study (Journal)	Study Type	Publication	Country	Study Groups	Study Period	Patient Selection
Kallen et al., 2010 [13]	Retrospective cohort	Peer-reviewed article	USA	N/A	July 2005–February 2008	All patients who underwent CTPA
Uehara et al., 2011 [16]	Retrospective cohort	Peer-reviewed article	Japan	N/A	January 2005–December 2006	Consecutive patients with high risk of PE based on symptoms and clinical data (low PaO2, low PaCO2, increased D-dimer, ECG, ultrasonography)
Shahir, K et al., 2013 [15]	Case-control	Peer-reviewed article	USA	Study: PE-positive CTPA Control: Randomly selected negative CTPA	2006–2008	Patients between 18–40 years old who underwent CTPA (first scan only if multiple). PE-positive scans selected first, then randomly selected controls (normal exam, non-PE related findings) chosen from same selection population.
Michalakis et al., 2014 [18]	Prospective cohort	Peer-reviewed article	Belgium	N/A	September 2010–July 2012	Consecutive patients who underwent CTPA based on clinical suspicion of PE
Shahir et al., 2015 [12]	Retrospective cohort	Peer-reviewed article	USA	N/A	2004–2012	All pregnant women who underwent CTPA
Atalay et al., 2011 [11] (Clin Rad)	Retrospective cohort	Peer-reviewed article	USA	N/A	January 2005–March 2006	Consecutive patients positive for acute PE
Atalay et al., 2011 [14] (J Cardiol Comput Tomogr)	Retrospective cohort	Peer-reviewed article	USA	N/A	February 2010–March 2010	Patients who presented with chest pain, hypoxemia, tachycardia, shortness of breath or variations of these as indication for their CTPA
Hendriks et al., 2019 [20]	Retrospective cohort	Peer-reviewed article	Netherlands	N/A	Not specified	Consecutive non-pregnant female patients who underwent CTPA
Patel et al., 2007 [23]	Retrospective cohort	Conference abstract	Not specified	N/A	Not specified	Not specified
Cowell & Sheridan, 2012 [22]	Retrospective cohort	Conference abstract	Not specified	N/A	January 2012–April 2012	Patients who underwent CTPA
Atweh et al., 2012 [21]	Retrospective cohort	Conference abstract	Not specified	N/A	2005–2011	All pediatric patients (0–25 years) with a PE-positive CTPA
Ho et al., 2019 [17]	Retrospective and prospective cohort	Conference abstract	England	A: Patients who underwent CTPA (retrospective; n = 153) B: Patients assessed for dose for standard and reduced scan coverage (prospective; n = 23)	A: Nov 2018 B: December 2018–January 2019	Patients who underwent CTPA
Chen et al., 2019 [19]	Retrospective cohort	Conference abstract	Australia	N/A	Not specified	Consecutive patients with a PE-positive CTPA

N/A = not applicable; PE = pulmonary embolism; CTPA = computed tomography pulmonary angiography.

**Table 2:** CTPA type, *z*-axis used and associated reduction in mean scan length and radiation dose in included studies.

Study (Journal)	CT Scan Type	Reduced Scan Window Used	Was the Optimal Scan Range Evaluated to Capture All PE Diagnoses/Filling Defects?	Reduction in Mean Scan Length (cm, % Reduction)	Reduction in Radiation Dose (%)
Kallen et al., 2010 [13]	64-row MDCT	Above the aortic arch to below inferior-most aspect of the heart	N	9.6 (37%)	N/A
Uehara et al., 2011 [16]	16-slice MDCT	Top of aortic arch to below the under surface of the heart	N	21.90%	22
Shahir et al., 2013 [15]	16-row and 64-row MDCT	Top of aortic arch to below the level of the heart	N	11 (42%); calculated based on 15 different consecutive patients who underwent CTPA	60; based on different 15 consecutive patients who underwent CTPA)
Michalakis et al., 2014 [18]	16-section and 64-row MDCT	10 cm scan length starting from the bottom of aortic arch	N	19.6 (52%)	69
Shahir et al., 2015 [12]	16-row and 64-row MDCT	Top of aortic arch to below the level of the heart	N	15 (42%)	71; calculated based on 36 consecutive non-pregnant adult patients who underwent CTPA
Atalay et al., 2011 [11] (Clin Rad)	16-row MDCT	A: 14.2 cm scan length centered 4.1 cm below the carina (capture at least one PE) B: Top of aorta to bottom of the heart	A: Y B: N	A: 11.7 (44%) B: 9.9 (38%)	N/A
Atalay et al., 2011 [14] (J Cardiol Comput Tomogr)	16-row and 64-row MDCT	14.2 cm scan length centered 4.1 cm below the carina (based on previous study which optimized scan length to capture all PE)	N	13.8 (49%)	N/A
Hendriks et al., 2019 [20]	A: 64-slice MDCT B: 2 × 128-slice DSCT C: 2 × 128-slice DSCT D: 2 × 192-slice DSCT	A-B: Lung apex to the top of the most caudal diaphragm	A-B: N	A: 33% B: 30% C: 30% D: 31%	A: 26% B: 25% C: 26% D: 23%
Patel et al., 2007 [23]	Not specified	Top of the aortic arch to below the heart	N	N/A	48
Cowell & Sheridan, 2012 [22]	Not specified	Superior aspect of the aortic arch to the inferior aspect of the heart	N	Not specified	Not specified
Atweh et al., 2012 [21]	Not specified	A: Patients without congenital heart disease: 14 cm scan length centered 3.5 cm below the carina (captures 100% of all filling defects) B: Patients with CHD: 8 cm scan length centered 5 cm below the carina (captures at least 1 filling defect)	A: Y B: Y	A: 20% B: 40%	Not specified
Ho et al., 2019 [17]	Not specified	Humeral heads to lung bases (excludes lung apices)	N	A & B: 14.9 cm (49.6%)	A: N/A B: 21
Chen et al., 2019 [19]	Not specified	14.7 cm length starting superiorly at the top of the aortic arch (no rationale provided)	N	N/A	N/A

**Table 3:** Study outcomes: yield of PE diagnoses, filling defects and other (non-PE) diagnoses with reduced CTPA scan coverage.

Study (Journal)	Total Number of CTPAs Analysed	Number of CTPAs Excluded	Reasons for Exclusion	Number of CTPAs Included	PE-Positive CTPAs (% of Included Scans)	Number of PE Diagnoses Missed with Reduced Scan Window (% of All PEs)	Number of Filling Defects Missed	Total Number of Other Findings	Number of Other Findings Missed with Reduced Scan Window (% of All Other Findings)
Kallen et al. 2010 [13]	1734	0	Not applicable	1734	295 (17)	0	Not specified	Not specified	Not specified
Uehara et al., 2011 [16]	75	0	Not applicable	75	75 (100)	0	2 (2.6%)	Not specified	Not specified
Shahir et al., 2013 [15]	878	678 (77%)	Suboptimal opacification of pulmonary arteries, compromised evaluation due to breathing artefact (n = 112); negative for PE; not randomly selected for control group	200	86 (43.7)	0	Not specified	392 (1.96 findings per scan)	7 (1.8)
Michalakis et al., 2014 [18]	253	6 (0.023%)	Poor arterial enhancement	247	57 (23.4)	1 (1.8; sub-segmental)	Not specified	343 (1.39 findings per scan)	48 (14)
Shahir et al., 2015 [12]	95	11 (8.64%)	Suboptimal contrast opacification; respiratory motion artefacts	84	2 (2.3; segmental [n = 2])	0	Not specified	76 (0.9 findings per scan)	4 (5.26)
Atalay et al., 2011 [11] (Clin Rad)	95	11 (8.64%)	Suboptimal contrast opacification; respiratory motion artefacts	84	2 (2.3; segmental [n = 2])	0	Not specified	76 (0.9 findings per scan)	4 (5.26)
Atalay et al., 2011 [14] (J Cardiol Comput Tomogr)	95	11 (8.64%)	Suboptimal contrast opacification; respiratory motion artefacts	84	2 (2.3; segmental [n = 2])	0	Not specified	76 (0.9 findings per scan)	4 (5.26)
Hendriks et al., 2019 [20]	95	11 (8.64%)	Suboptimal contrast opacification; respiratory motion artefacts	84	2 (2.3; segmental [n = 2])	0	Not specified	76 (0.9 findings per scan)	4 (5.26)
Patel et al., 2007 [23]	95	11 (8.64%)	Suboptimal contrast opacification; respiratory motion artefacts	84	2 (2.3; segmental [n = 2])	0	Not specified	76 (0.9 findings per scan)	4 (5.26)
Cowell & Sheridan, 2012 [22]	200	161 (80.5%)	Negative for PE	39	39 (100)	0	Not specified	Not specified	3 patients with other findings (number of missed findings not specified)
Atweh et al., 2012 [21]	Not specified	Not specified	Negative for PE	45	45 (100)	A: 0 B: 0	A: 0 B: Not specified	Not specified	Not specified
Ho et al., 2019 [17]	A: 153 B: 23	Not specified	Not specified	A: 153 B: 23	A: 29 (19) B: Not specified	0	Not specified	Not specified	Not specified
Chen et al., 2019 [19]	200	Not specified	Not specified	200	200 (100)	1 (0.5; sub-segmental)	2 (1%)	Not specified	Not specified

PE = pulmonary embolism.

## Supplementary Materials

The following are available online at https://www.mdpi.com/article/10.3390/diagnostics11122179/s1, Supplementary Table S1: Search strategy performed in MEDLINE and EMBASE, Supplementary Table S2: Risk of bias assessment tool, Supplementary Table S3: Risk of bias assessment, Supplementary Table S4: List of excluded studies.
