# Supplementary material for: The Effect of Limiting the Scan Range of Computed Tomography Pulmonary Angiography (to Reduce Radiation Exposure) on the Detection of Pulmonary Embolism: A Systematic Review
Source: Diagnostics (Basel). 2021 Nov 24;11(12):2179. doi: 10.3390/diagnostics11122179 (PMC8700432; doi:10.3390/diagnostics11122179)
Supplement: Supplementary file 1 [file diagnostics-11-02179-s001.zip › diagnostics-1455597-supplementary.pdf]

The effect of limiting the scan range of computed tomography pulmonary angiography (to reduce radiation exposure) on the detection of pulmonary embolism: A systematic review

Amayar Zaw,[1,2] Rebecca Nguyen,[1,2] Leon Lam,[3] Anthony Kaplan,[2,3] Claudia C. Dobler[1,2,4,5*]

1 Department of Respiratory and Sleep Medicine, Liverpool Hospital, Sydney, Australia
2 South Western Sydney Clinical School, University of New South Wales, Sydney, Australia
3 Department of Radiology, Liverpool Hospital, Sydney, Australia
4 The George Institute for Global Health, Sydney, Australia
5 Woolcock Institute of Medical Research, The University of Sydney, Sydney, Australia
* Correspondence: c.dobler@unsw.edu.au; Tel.: +61 2 8738 3000

Supplementary Table S1: Search strategy performed in MEDLINE and EMBASE

#	Search strategy
1	Pulmonary embolism/dg
2	Pulmonary emboli*
3	Computed, x-ray tomography/mt
4	CTPA or computed tomography pulmonary angiogra*
5	1 or 2
6	3 or 4
7	5 and 6
8	Z-axis or scan length or acquisition length or CTPA length or scan cover* or CTPA cover* or anatomic* cover* or anatomic* window* or scan redu* or dose or dosage
9	7 and 8
10	9
12	Remove duplicates from 10

Supplementary Table S2: Risk of bias assessment tool

Criteria		Scoring	
Excluded scans			
Of the number of CTPAs analyzed, a significant proportion (≥20%) were excluded for poor imaging quality (suboptimal contrast, motion artefact)	a.<br>b.<br>c.	<20%**<br>≥20%<br>Not specified	3 points<br>0 point<br>0 point
Outcome			
Expert(s) reporting CTPA blinded to original CTPA report	a.<br>b.	Yes* points<br>No/not specified	2<br>0 point
Years of experience of expert (s) reporting (if ≥2 experts, scored based on the most experienced investigator)	a.<br>b.	≥5 years<br><5 years/not specified	1 point<br>0 point
Two independent experts assessing	a.<br>b.	Yes* point<br>No/not specified	2<br>0 point

Total score – maximum of 8; low risk of bias score ≥6; high risk of bias score <6.

Supplementary Table S3: Risk of bias assessment

**Study (Journal)**	**Excluded scans**	**Outcome**			**Total points**
	**Of the number of CTPAs analysed, a significant proportion (≥20%) were excluded for poor imaging quality (suboptimal contrast, motion artefact)**	**Radiologist(s) reporting CTPA blinded to original CTPA report**	**Years of experience of radiologist(s) reporting (if ≥2 experts, scored based on the most experienced investigator)**	**Two independent radiologists assessing**	
Peer-reviewed papers					
Kallen et al. 2010	0<br>(not specified)	0<br>(not blinded)	**1**<br>(>10 years)	0<br>(no)	**1**
Uehara et al. 2011	0<br>(not specified)	0<br>(not specified)	0<br>(not specified)	**2**<br>(yes)	**2**
Shahir et al. 2013	**3**<br>(12.8%)	**2**<br>(blinded)	**1**<br>(>5 years)	**2**<br>(yes)	**8**
Michalakis et al. 2014	**3**<br>(2.3%)	**2**<br>(blinded)	**1**<br>(>12 years; >26 years)	**2**<br>(yes)	**8**
Shahir et al. 2015	**3**<br>(11.6%)	**2**<br>(blinded)	0<br>(>4 years)	**2**<br>(yes)	**7**
Atalay et al. 2011 (Clin Rad)	0<br>(not specified)	0<br>(not specified)	**1**<br>(>6 years)	**2**<br>(yes)	**3**
Atala et a.l 2011 (J Cardiol Comput Tomogr)	0<br>(not specified)	0<br>(not specified)	**1**<br>(>10 years)	**2**<br>(yes)	**3**
Hendriks et al. 2019	0<br>(not specified)	0<br>(not specified)	**1**<br>(>45 years)	0<br>(not specified)	**1**
Conference abstracts					
Patel et al. 2007	0<br>(not specified)	0<br>(not specified)	0<br>(not specified)	0<br>(not specified)	**0**
Cowell& Sheridan 2012	0<br>(not specified)	0<br>(not specified)	0<br>(not specified)	0<br>(not specified)	**0**
Atweh et al. 2012	0<br>(not specified)	0<br>(not specified)	0<br>(not specified)	0<br>(not specified)	**0**
Ho et al. 2019	0<br>(not specified)	0<br>(not specified)	0<br>(not specified)	0<br>(not specified)	**0**
Chen et al. 2019	0<br>(not specified)	0<br>(not specified)	0<br>(not specified)	0<br>(not specified)	**0**

Scoring: low bias risk, total score ≥6; high bias risk, total score <6

Supplementary Table S4: List of excluded studies.

Study Authors	Study Title	Study Goal	Study Design	Study Population	Reason for exclusion
Aldosari et al. 2019[1]	Patient-specific 3D printed pulmonary artery model with simulation of peripheral pulmonary embolism for developing optimal computed tomography pulmonary angiography protocols.	Utilising a 3D printed pulmonary model for simulation of small thrombus for development of optimal CTPA protocols.	Sample case of patient with diagnosed PE was retrospectively chosen and a patient-specific 3D printed model was created. Thrombus was simulated in peripheral arteries and model was scanned with different CTPA protocols. Image quality and thrombus visualisation assessed.	Single patient was chosen as sample image. Reasoning not specified.	Simulation model not relevant to the study.
Aldosari et al. 2019[2]	Optimization of computed tomography pulmonary angiography protocols using 3D printed model with simulation of pulmonary embolism.	Investigating optimimal CTPA protocols for detection of PE using 3D printed models of pulmonary arteries.	Sample case of patient with diagnosed PE was retrospectively chosen and a patient-specific 3D printed model was created. Thrombus was simulated in main pulmonary aerteries and model was scanned with different CTPA protocols. Image quality and thrombus visualisation assessed.	Single patient was chosen as sample image. Reasoning not specified.	Simulation model not relevant to the study.
Nania et al. 2018[3]	CTPA protocol optimisation audit: challenges of dose reduction with maintained image quality.	To assess CTPA dose and image quality in a single teaching hospital for optimisation of dosage whilst preserving image quality.	Audit of random patients undergoing CTPA during a 1-week period. Dose-length product, patient size and quality parameters recorded and independent image quality evluation carried out by 3 specialist cardiothoracic radiologists.	Randomly selected patients undergoing CTPA.	Evaluation of radiation dose and image quality not the scan range.
Armstrong et al. 2017[4]	Survey of UK imaging practice for the investigation of pulmonary embolism in pregnancy.	Determine the utilisation of CTPA and V/Q scans in the UQ and assess diagnostic qualities in assessment of suspected PE in pregnant women.	Retrospective electronic questionnaire of radiologists across 24 different sites. Collected data in regards to choice of imaging technique, radation dose, technical adequacy, weeks’	Thoracic radiologists across the UK.	Study type (survey) is not relevant to the study.

Study Authors	Study Title	Study Goal	Study Design	Study Population	Reason for exclusion
			gestation, presenting symptoms and further management with indeterminent imaging.		
Martilotti et al. 2013[5]	Dose of reduced z-axis length of computed tomography angiography (CTA) of the chest for pulmonary embolism using 64-detector rows and adaptive iterative reconstruction techniques.	Comparison of reduced z-axis length CTPA compared with adaptive iterative reconstruction techniques in the diagnosis of PE.	Retrospective study comparing scan length reduction and effective dose reduction between reduced z-axis and standard length CTPA scans. Main outcome of interest was mean effective dose receieved.	All emergency room patients under 50 years of age, with no significant comorbidities, who underwent CTPA with reduced z-axis protocol between February 2011 and an unspecified end period. Control group consisted of consective patients from same time period of patient criteria who underwent CTPA with standard length protocol.	Evaluation of radiation dose not diagnostic yield of PE.
Lee et al. 2012[6]	Multidetector CT pulmonary angiography: Value of multiplanar reformation images in detecting pulmonary embolism in children.	Evaluate thromboembolic risk factors for PE detected by CTPA in children to determin whether such information could be used for appropriate CTPA use in the paediatric population.	Retrospective study of 227 paediatric patients who underwent CTPA. Age, sex, referral setting and D-dimer results and seven possible risk factors compared between patients with and without PE. Multiple logistic regression modelling used to identify independent risk factors of PE.	All paediatric patients who underwent CTPA with suspicion of PE based on clinical signs and symptoms between July 2004 and March 2011.	Paediatric risk factors for PE not relevant to the study.
Lee et al. 2011[7]	Pulmonary MDCT (multidetector CT) angiography: value of multiplanar reformatted images in detecting pulmonary embolism in children.	Determine whether the use of multiplanar reformated (MPR) MDCT images in the diagnosis of PE in children affects reader performance parameters and adds diagnostic value comapred with axial MDCT images	Retrospective study of 60 paediatric patient's images. Four independent radiologits reviewed axial and MPR MDCT images and evaluated image quality, presence of PE, confidence level, interpretation time and diagnostic value of	Paediatric patients who underwent pulmonary MDCT angiography with suspicion of PE based on clinical signs and symptoms.	Diagnostic yield of PE with reduced z-axis not assessed.

Study Authors	Study Title	Study Goal	Study Design	Study Population	Reason for exclusion
		alone.	MPR MDCT images.		
Bauer et al. 2011[8]	Dose and image quality at CT pulmonary angiography-comparison of first and second generation dual-energy CT and 64-slice CT.	To compare the dose and image quality of 64-slice, first and second generation dual-energy CT for CTPA.	Retrospective study of four different groups of patients who underwent CTPA on a first generation or second generation dual-energy CT for CTPA. CTDIvol, dose-length product, background noise, thorax diameter and attenuation in pulmonary trunk were compared.	Every patient referred for CTPA for suspected CTPA between August 2007 and June 2010.	Comparing CTPA dose and image quality not relevant to the study
Espinosa et al. 2010[9]	Clinical utility of multiplanar reformation in pulmonary CT angiography.	Determine whether multi-planar or axial view for CTPA interpretation for suspected PE alters diagnostic confidence, accuarcy and interpretation time.	Retrospecitve study of CTPA images by 5 independent radiologists. Axial-only images analysed first, then several months later, multi-planar display was analysed. For each image, ratings were given in regard to overall determination of PE, diagnostic confidence, image quality and time taken to interpret.	50 patients who underwent CTPA (with 64-MDCT scanner) between March 7, 2006, to March 23, 2006.	Reduced z-axis not assessed.
Litmanovich et al. 2009[10]	Dose reduction in computed tomographic angiography of pregnant patients with suspected acute pulmonary embolism.	To quantify the effect of reduced-dose CTPA protocol on radiation dose and image quality in pregnant patients compared with standard protocol.	Retrospective study comparing low-dose CTPA images in the pregnant women group with standard dose CTPA in control group. Attenuation measurements, signal-to-noise ratio, radiation dose and image quality were assessed and compared.	Pregnant women referred for CTPA by the emergency department from July 2006 to July 2007.<br>Control group consisted of non-pregnant women within same timeframe.	Reduced z-axis not assessed.

References

1. Aldosari, S.; Jansen, S.; Sun, Z. Optimization of computed tomography pulmonary angiography protocols using 3D printed model with simulation of pulmonary embolism. *Quantitative Imaging in Medicine and Surgery* **2019**, *9*, 53-62.
2. Aldosari, S.; Jansen, S.; Sun, Z. Patient-specific 3D printed pulmonary artery model with simulation of peripheral pulmonary embolism for developing optimal computed tomography pulmonary angiography protocols. *Quantitative Imaging in Medicine and Surgery* **2019**, *9*, 75-85.
3. Nania, A.; Weir, A.; Weir, N.; Ritchie, G.; Rofe, C.; Van Beek, E. CTPA protocol optimisation audit: challenges of dose reduction with maintained image quality. *Clinical Radiology* **2018**, *73*, 320.e321-320.e328.
4. Armstrong, L.; Gleeson, F.; Mackillop, L.; Mutch, S.; Beale, A. Survey of UK imaging practice for the investigation of pulmonary embolism in pregnancy. *Clinical Radiology* **2017**, *72*, 696-701.
5. Martillotti, J.; Silva, N.; Chhabra, J.; Molstrom, C.; Coughlin, R.; O'Loughlin, M.; Coughlin, B. Dose of reduced z-axis length of computed tomography angiography (CTA) of the chest for pulmonary embolism using 64-detector rows and adaptive iterative reconstruction techniques. *Emergency Radiology* **2013**, *20*, 39-44.
6. Lee, E.; Zucker, E.J.; Tsai, J.; Tracy, D.A.; Zurakowski, D.; Boiselle, P.M. Multidetector CT pulmonary angiography: Value of multiplanar reformation images in detecting pulmonary embolism in children. *Pediatric Radiology* **2012**, *2)*, S269.
7. Lee, E.Y.; Zucker, E.J.; Tsai, J.; Tracy, D.A.; Cleveland, R.H.; Zurakowski, D.; Boiselle, P.M. Pulmonary MDCT angiography: value of multiplanar reformatted images in detecting pulmonary embolism in children. *AJR. American Journal of Roentgenology* **2011**, *197*, 1460-1465.
8. Bauer, R.W.; Kramer, S.; Renker, M.; Schell, B.; Larson, M.C.; Beeres, M.; Lehnert, T.; Jacobi, V.; Vogl, T.J.; Kerl, J.M. Dose and image quality at CT pulmonary angiography-comparison of first and second generation dual-energy CT and 64-slice CT. *European Radiology* **2011**, *21*, 2139-2147.
9. Espinosa, L.A.; Kelly, A.M.; Hawley, C.; Inampudi, R.; Chughtai, A.; Agarwal, P.; Khalatbari, S.; Myles, J.; Kazerooni, E. Clinical utility of multiplanar reformation in pulmonary CT angiography. *AJR. American Journal of Roentgenology* **2010**, *194*, 70-75.
10. Litmanovich, D.; Boiselle, P.M.; Bankier, A.A.; Kataoka, M.L.; Pianykh, O.; Raptopoulos, V. Dose reduction in computed tomographic angiography of pregnant patients with suspected acute pulmonary embolism. *Journal of Computer Assisted Tomography* **2009**, *33*, 961-966.
